# Breathable Electronic Skins for Daily Physiological Signal Monitoring

**DOI:** 10.1007/s40820-022-00911-8

**Published:** 2022-08-09

**Authors:** Yi Yang, Tianrui Cui, Ding Li, Shourui Ji, Zhikang Chen, Wancheng Shao, Houfang Liu, Tian-Ling Ren

**Affiliations:** 1 School of Integrated Circuit, and Beijing National Research Center for Information Science and Technology, Tsinghua University, Beijing, 100084 People’s Republic of China; 2 Center for Flexible Electronics Technology, Tsinghua University, Beijing, 100084 People’s Republic of China

**Keywords:** Electronic skin, Breathable, Physiological signal monitoring, Wearable systems

## Abstract

With the aging of society and the increase in people’s concern for personal health, long-term physiological signal monitoring in daily life is in demand. In recent years, electronic skin (e-skin) for daily health monitoring applications has achieved rapid development due to its advantages in high-quality physiological signals monitoring and suitability for system integrations. Among them, the breathable e-skin has developed rapidly in recent years because it adapts to the long-term and high-comfort wear requirements of monitoring physiological signals in daily life. In this review, the recent achievements of breathable e-skins for daily physiological monitoring are systematically introduced and discussed. By dividing them into breathable e-skin electrodes, breathable e-skin sensors, and breathable e-skin systems, we sort out their design ideas, manufacturing processes, performances, and applications and show their advantages in long-term physiological signal monitoring in daily life. In addition, the development directions and challenges of the breathable e-skin are discussed and prospected.

## Highlights

- This work reviews the development of breathable electronic skins (e-skins) for daily physiological signal monitoring in recent years.
- The design methods, preparation processes, and performances of typical breathable e-skin electrodes, breathable e-skin sensors, and breathable e-skin systems developed these years are systematically introduced and discussed.
- This review analyzes the challenges that breathable e-skins may face in daily health monitoring and puts forward the possible development directions of breathable e-skins.

## Graphical abstract

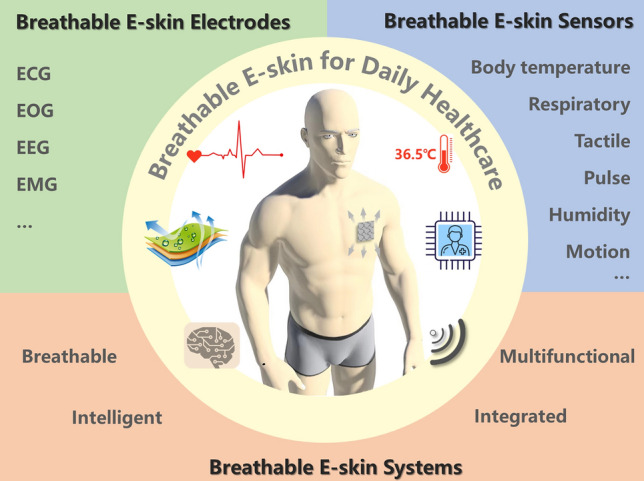

## Introduction

In recent years, electronic skins (e-skins) for daily health monitoring applications have achieved rapid development due to their advantages in daily physiological signal monitoring: (1) The flexible substrate of e-skin can attach to human skin with high conformability, which helps to collect higher quality physiological signals [1–3]. (2) The e-skins have developed from single-mode to multifunction [4, 5], and their outstanding detection performances show their great potential in medical level applications [6]. (3) Due to their high system integration potential, e-skins are becoming systematic to achieve both physiological signal acquisition and diagnosis, which is suitable for daily health management like a “portable doctor” [7, 8]. Thus, many high-performance e-skins for wearable daily physiological signal monitoring have been developed [9–11]. Meanwhile, the demand for daily and long-term physiological signal monitoring promotes higher requirements for the development of e-skin.

With the increasing need for long-term and comfortable physiological signal monitoring through e-skin, breathable (air-permeable) e-skins have attracted particular attention in recent years [12–15]. The breathable e-skin can avoid the accumulation of sweat and greatly improve the comfort of long-term wear [16]. Based on the types of monitoring signals, the breathable e-skins for daily long-term physiological signal monitoring can be divided into two main categories. One is the breathable e-skin electrode for electrophysiological signal monitoring [17–20], including the electrocardiograph (ECG) e-skin electrode, electro-oculography (EOG) e-skin electrode, electromyography (EMG) e-skin electrode, electroencephalograph (EEG) e-skin electrode, etc. The other is breathable e-skin sensors [21–25], which are used to detect both physical physiological signals (pules, breath sound, blood pressure, body temperature, etc.) and chemical physiological signals (glucose, ethanol, electrolytes, etc.). Besides, there are also multifunctional breathable e-skins for detecting multiple physiological signals [5]. According to different physiological signals or multiple physiological signals monitoring requirements, the functional materials, structures, and fabricating processes of breathable e-skins are various. Furthermore, considering the requirements of detecting and analyzing physiological signals, the system integration requirements of detecting and analyzing physiological signals, the e-skins for daily health monitoring are becoming more multifunctional, more integrated, and more intelligent to realize a doctor-like daily healthcare system.

In this review, recent advances in breathable e-skin electrodes, sensors, and systems for daily physiological signal monitoring are introduced, respectively. Their design methods, functional materials, structures, fabricating processes, and performances are systematically discussed. Then, the typical multifunctional, highly integrated, and intelligent e-skin systems are shown, which are competent for daily independent work and long-term in situ sensing-processing-diagnosis of physiological signals in daily life. Finally, we discuss the challenges that breathable e-skins may face in daily health monitoring and conclude with comments on their future directions.

## Breathable e-Skin Electrodes

Since most electrophysiological signal monitoring requires direct contact between the conductive material and human skin to record the signals with high-quality and long-term stability, some important factors should be taken into account. First, the conductive materials should have high conductivity and stable contact with human skin to record high-quality signals. Second, the substrate materials should have good breathability and biocompatibility to avoid skin irritations and conform to human skin. Third, the electrode should possess stable physical and chemical properties for daily long-term use. Fourth, as electrodes for daily health monitoring, their process complexity and material costs need to be considered. In this section, according to types of monitoring physiological signals, we introduce the development of the typical breathable e-skins in the applications of monitoring ECG, EOG, EMG, EEG, and so on, respectively. The preparation methods and characteristics of these breathable e-skin electrodes are also described emphatically.

### Breathable ECG e-Skin Electrodes

Cardiovascular disease is a major threat to global human health, which can be prevented and diagnosed by a simple and effective solution, which is ECG monitoring [26]. However, because of the short monitoring time of the static ECG acquisition in hospitals and the relatively fixed physical examination time, misdiagnosis or missed diagnosis may occur [27]. At present, long-term ECG monitoring is an important way to monitor cardiovascular health [28]. Accordingly, it is necessary to develop breathable, durable, and comfortable e-skin electrodes for daily long-term ECG monitoring. The breathable ECG e-skin electrode has developed rapidly in recent years due to its suitability for long-term skin wearing and high-quality signal acquisition.

**Fig. 1:**
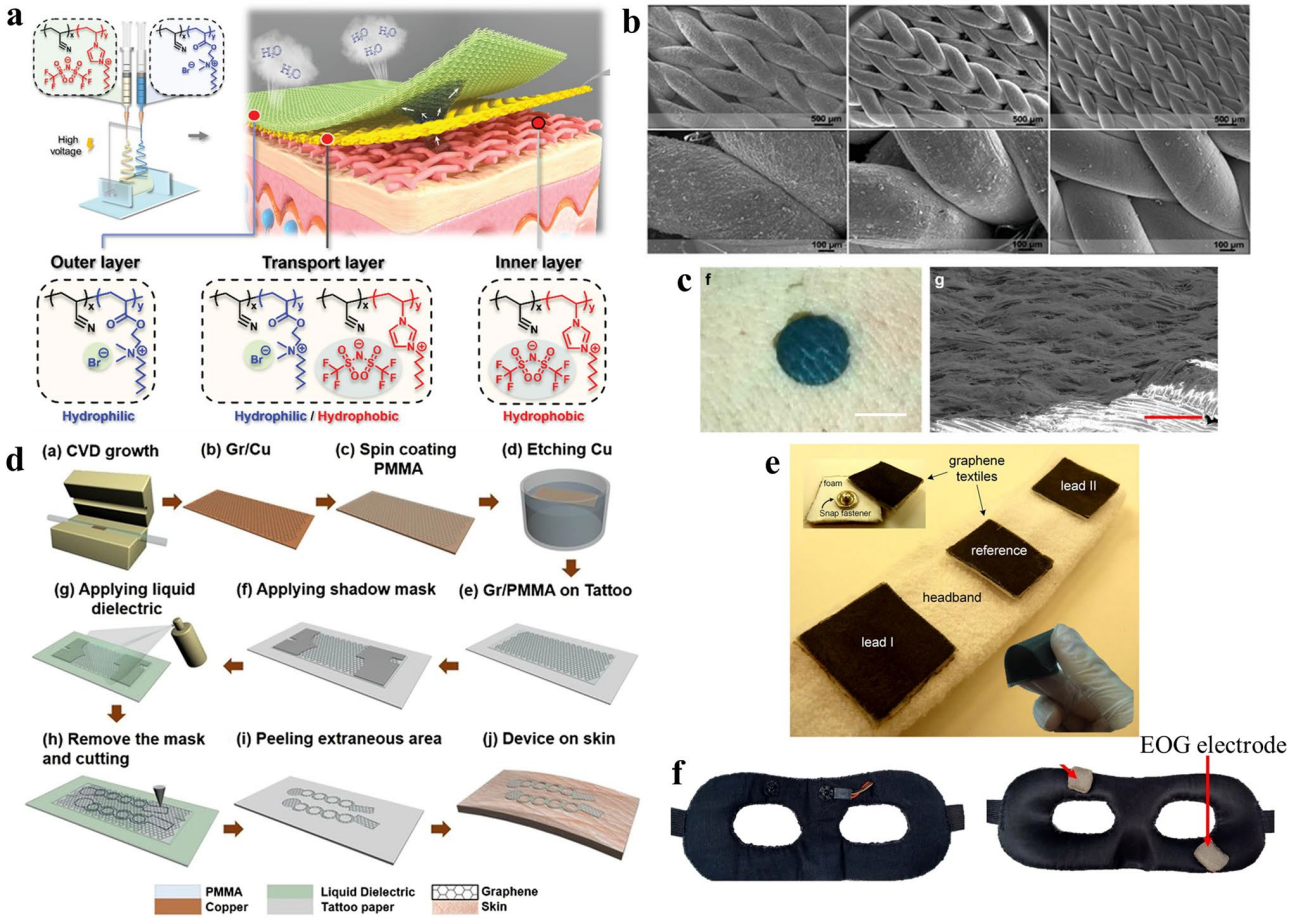
**a** Schematic illustration of the dual-gradient poly (ionic liquid) nanofiber-based e-skin electrode. Reproduced with permission from Ref. [29]. Copyright 2021 Wiley. **b** SEM images of textile e-skin made from different conductive elastomeric fibers. Reproduced with permission from Ref. [30]. Copyright 2022 Wiley. **c** Photograph and SEM images of the substrate-free e-skin on the human skin. The scale bars represent 1 cm and 200 μm, respectively. Reproduced with permission from Ref. [31]. Copyright 2020 American Chemical Society. **d** Fabrication process of the imperceptible graphene-based EOG e-skin electrode. Reproduced with permission from Ref. [36]. Copyright 2018 Springer Nature. **e** EOG headband with graphene textile e-skin electrodes. Reproduced with permission from Ref. [19]. Copyright 2019 IOPscience. **f** EOG e-skin integrated with an eye mask. Reproduced with permission from Ref. [38]. Copyright 2021 Springer

To avoid the influence of sweat on the comfort of wearing, electrode stability, and signal quality of e-skin electrodes, Zheng et al. proposed a breathable, moisture-wicking, and antibacterial e-skin [29]. Unlike traditional breathable fiber-based e-skins, which are either hydrophilic to moisturize the skin, or hydrophobic and unable to expel moisture, the dual-gradient poly (ionic liquid) nanofiber membrane with asymmetric wettability can continuously transport sweat from the skin side to the air side. This ECG e-skin consisted of a hydrophilic outer layer, a transport layer, and a hydrophobic inner layer with gradients of both hydrophilicity and pore size (Fig. 1a). The multiwalled carbon nanotubes were sprayed between the inner layer and the transport layer as a conductive layer to ensure that the e-skin had good conductance to ECG signals. Besides, the poly (ionic liquid) interacted with the negatively charged bacterial surface and broke the bacterium. The acquired e-skin presented breathable, anti-bacteria, and no sweat accumulation. Compared with commercial Ag/AgCl gel electrodes, the e-skin electrode had good long-term monitoring performance and could not cause accumulation of sweat and skin allergy, suitable for the daily ECG monitoring application.

The mature garment industry has made clothing more durable, breathable, and comfortable. If the ECG e-skin electrode can adopt the woven structure like clothing, it can not only meet the requirements of breathability and durability but also be suitable for integration with daily clothing to achieve long-term wearable health monitoring. Eskandarian et al*.* used conductive elastomeric filaments to realize a three-dimensional (3D) conductive e-skin with an industrial-scale knitting machine [30]. As shown in Fig. 1b, the textile e-skin had a 3D textile structure that ensured a dense conductive network, while its melt-spun fibers had good flexibility (strain > 200%) and breathability (about 20 g m^−2^ h^−1^ water vapor permeability). Besides, the e-skin could realize 24 h continuous ECG monitoring in daily living conditions, demonstrating its durability and stability in complex usage environments. Moreover, the realized textile ECG e-skin could be integrated into the garments seamlessly and its performance remained stable after 30 times washing. The textile-based breathable e-skin meets the needs of long-term ECG monitoring in daily life and has the potential of forming smart clothing for daily health monitoring.

At present, most wearable ECG electrodes have substrates. Although some of their substrates are breathable, their existence limits the evaporation of sweat and decreases the flexibility and comfort of e-skins. To realize an e-skin with excellent breathability while maintaining good mechanical properties, Qiao et al*.* developed a substrate-free e-skin based on laser-scribed graphene [31]. The e-skin was prepared by laser-reduced graphene oxide which was drop cast on a transfer paper. By using a water-soluble sacrificial layer, the laser-scribed and substrate-free graphene layer could be separated from the unreduced graphene oxide and transferred onto the human skin (Fig. 1c). Compared with e-skins with substrates, the substrate-free e-skin has good breathability (it hardly affects skin perspiration), high flexibility (100% strain range), and low resistance (1 Ω in the vertical direction). Most significantly, the substrate-free e-skin can attach to the uneven human skin surface conformably, which greatly decreases the skin–electrode contact impedance (5.6 kΩ at 1 kHz). Because of its good fit with skin, it is not easy to move relative to the skin, which makes the collected ECG signal has good quality. After combined with a flexible signal processing module and a convolutional neural network, the ECG e-skin can be used to detect the human ECG signal in real-time. Moreover, the substrate-free e-skin can also be used as a strain sensor to detect a variety of physical physiological signals with high sensitivity.

### Breathable EOG e-Skin Electrodes

EOG reflects the electrophysiological signals between the cornea and the retina of human eyes [32], which is widely used in the research of brain science, mental disease diagnosis, sleep, etc. [33]. Although EOG, EEG, and EMG can all be used in the diagnosis of neurological abnormalities, the EOG has dominant advantages including: (1) The analysis of the EOG signals is easier because the EOG has a linear relationship to eye movements in a certain range. (2) EOG signals are more stable than EEG and EMG signals [34]. At present, however, many state-of-the-art EOG electrodes are bulky, airtight, rigid, and uncomfortable to wear [35]. As EOG electrodes are applied to the delicate skin around the eyes, the EOG electrodes are expected to be flexible, air-permeable, and soft to avoid skin damage, fatigue, or irritation, which makes them good candidates for EOG electrodes. Besides, flexible e-skin electrodes can conform to the surface of human skin, so they can monitor the EOG signals with low impedance, high signal-to-noise ratios (SNR), and high quality. In this section, we will introduce some advanced breathable EOG e-skin electrodes and demonstrate their advantages in the daily long-term monitoring of EOG signals.

Facing the lack of high-performance breathable EOG electrodes, Ameri et al*.* developed an imperceptible and breathable tattoo-like graphene e-skin [36]. The graphene was fabricated by chemical vapor deposition (CVD) and transferred onto a piece of commercial tattoo paper with the help of poly (methyl methacrylate) (PMMA). After being cut into an electrode shape, the graphene layer was transferred to the human skin by a tattoo paper (Fig. 1d). The realized ultrathin, ultrasoft, and transparent tattoo-like EOG e-skin was substrate-free and could be laminated around the eyes without using any adhesives. Besides, they did not constrain eye movements or facial expressions. Due to their good contact with the skin, the SNR of the tattoo-like e-skin was 15.22 dB, which was much higher than the commercial Ag/AgCl gel electrodes in detecting EOG signals. Moreover, the EOG e-skin realized a 4° high angular resolution of eye movement. In their experiment, the high-precision EOG signals acquired by the breathable e-skin electrode were successfully used to control a drone in real-time, which showed its high performance in detecting long-term EOG signals and great potential in human–robot interaction.

As an ideal carrier of long-term EOG monitoring electrodes, the headband is a suitable daily wearable platform. Besides, the head-worn fabric itself can act as a breathable substrate. Inspired by the headband, Golparvar et al*.* proposed a textile-based EOG e-skin that was directly weaved into a headband [19, 37]. The conductive textiles were fabricated from graphene-coated commercial fabrics, which were low-cost, durable, and scalable such as nylon and cotton. The graphene oxide suspension prepared by a modified Hummer’s method was dip-coated on the textiles. After being treated with the reducing agent, the graphene oxide particles were reduced and a conductive graphene network was formed on fabrics (Fig. 1e). The breathable EOG e-skin revealed an excellent correlation of 91.3% against the commercial Ag/AgCl EOG gel electrode during the long-term EOG monitoring. With good biocompatibility, the e-skins were integrated into a fabric headband and realized auto-detection of multiple eye movements, which anticipated their potential in EOG-based human–machine interfaces.

EOG monitoring also shows healthcare and medical value in sleep monitoring. For long-term sleep monitoring, the EOG electrode should be soft, lightweight, and breathable to minimize interference with the wearer’s normal sleep activities. To realize a daily-use EOG e-skin for high-comfort and real-time EOG signal monitoring, Hsieh et al*.* realized a breathable soft-fabric-based EOG e-skin [38]. The conductive fabrics with 20% silver and 80% polyamide formed a dense and breathable conductive network. Besides, a sponge package was used to relieve the contact pressure of the e-skin electrode on the skin around the eye while ensuring its breathability. The sponge package also ensured the durability and stability of the e-skin in long-term EOG monitoring. By integrating the breathable e-skin into an eye mask, the EOG e-skin with only 9 g weight and high flexibility realized convenient signal monitoring during sleep, and even continuous 24 h EOG recording. By cooperating the e-skin with the edge-computing module, the acquired EOG e-skin system gave a real-time sleep quality score, indicating that the soft-fabric-based e-skin was promising in daily high comfort EOG monitoring.

### Breathable EMG e-Skin Electrodes

Monitoring epidermal EMG is an effective way to reflect human muscle activity [39], which has important applications in guiding daily sports health, rehabilitation training, neuro-medicine, etc. [40]. Epidermal e-skins are considered ideal electrodes for daily EMG signal monitoring due to their skin conformability and excellent detecting capabilities [41]. Although many kinds of EMG e-skins have been developed these years. However, most of them are designed without considering breathability. The lack of breathability is a particularly serious defect for the epidermal EMG electrodes since many of their application scenarios of them in daily life are related to sports. Such an application environment with a large amount of sweat and severe skin deformation is likely to lead to the accumulation of sweat in the electrode–skin interface, which may cause irritation or electrode damage. Moreover, the accumulation of sweat not only easily leads to skin allergy or uncomfortable wearing but also greatly affects the quality of collected EMG signals [42]. To make EMG e-skin electrodes suitable for daily use, it is necessary to improve their breathability and durability while ensuring their electrical performance. In this section, we will introduce typical e-skin electrodes for EMG monitoring which have preliminary solved the above problems.

**Fig. 2:**
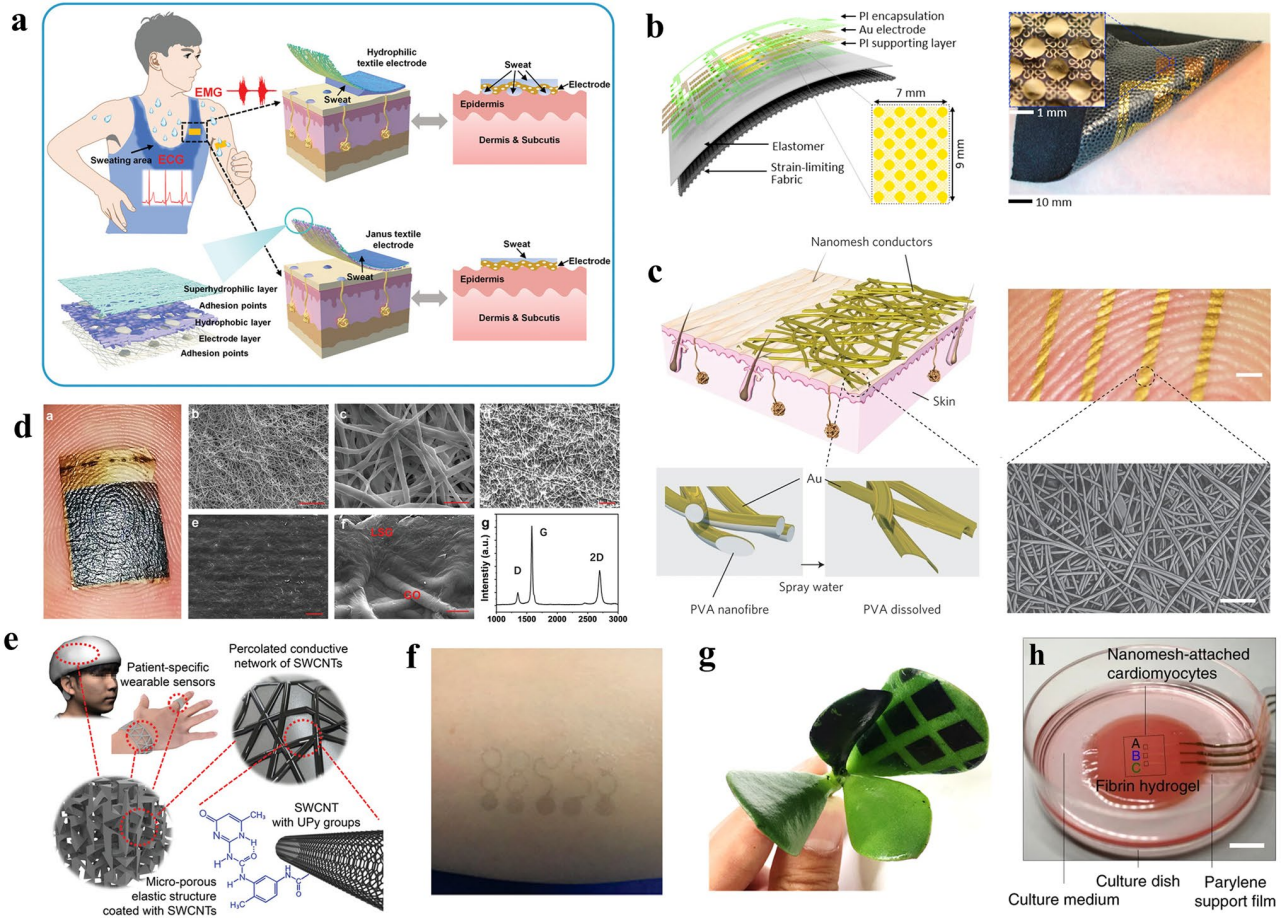
**a** The all-nanofiber-based e-skin electrode with directional sweat transport properties. Reproduced with permission from Ref. [43]. Copyright 2022 Wiley. **b** SEM images of textile electrodes made from different conductive elastomeric fibers. Reproduced with permission from Ref. [44]. Copyright 2020 Elsevier. **c** The Au-PVA nanomesh e-skin. Reproduced with permission from Ref. [45]. Copyright 2017 Springer Nature. **d** LSG-PU nanomesh e-skin electrode. Reproduced with permission from Ref. [5]. Copyright 2022 Wiley. **e** Porous elastomer-carbon nanotube e-skin electrode based on 3D-printed sugar scaffold. Reproduced with permission from Ref. [49]. Copyright 2019 Wiley. **f** Graphene tattoo-like EEG electrode. Reproduced with permission from Ref. [13]. Copyright 2017 American Chemical Society. **g** The vapor-printed breathable electrodes on plants. Reproduced with permission from Ref. [50]. Copyright 2020 AAAS. **h** Nanomesh e-skin is detecting ECG signals of cardiomyocytes. Reproduced with permission from Ref. [51]. Copyright 2019 Springer Nature

Aiming at the problem of sweat accumulation caused by airtight epidermal EMG electrodes, Yang et al. proposed an all-nanofiber-based e-skin that has directional sweat transport properties [43]. As shown in Fig. 2a, the designed multilayer e-skin consisted of superhydrophilic hydrolyzed-polyacrylonitrile (HPAN), polyurethane (PU), and silver nanowires (AgNWs). The HPAN layer, the medical adhesive layer, and the PU layer were all fabricated by electrospinning to form an all-porous breathable structure. The AgNWs were dispersed on these fibers through vacuum filtration to form a dense conductive network. With a hydrophilic–hydrophobic gradient structure, the e-skin could evaporate the sweat from the electrode–skin interface in 5 s with a water vapor permeability of 1748.09 g m^−2^ d^−1^, which was higher than the vapor transmission rate of the human skin. The sheet resistance of the electrode was ultra-low (4.3 Ω sq^−1^) and could be easily changed by adjusting the quantity of the AgNW. Besides, with a high adhesion force between the e-skin and human skin (430.87 mN cm^−1^) and high flexibility (strain > 500%), the all-fiber-based e-skin was suitable for daily long-term EMG monitoring on the sweaty human skin.

Monitoring epidermal EMG signals is a useful way of collecting feedback information in rehabilitation therapy. To accurately and continuously record muscle responses through EMG, the e-skin electrodes should be suitable for long-term wear with high detecting precision. Kwon et al*.* proposed a breathable strain-limiting e-skin with a 16-channel EMG electrode array to meet the demand of monitoring Hoffmann’s reflex on patients with spinal cord injury through EMG [44]. As shown in Fig. 2b, the multilayer e-skin consisted of a 16-channel gold electrode array, a polyimide supporting layer, a polyimide packaging layer, and a porous elastomer-fabric substrate that could attach to the human skin with high conformability. With high conductivity and biocompatibility, the electrode array realized high-fidelity recording of EMG signals from the forearm. Besides, the breathable e-skin showed non-cytotoxic and lower allergic reactivity than the commercial Ag/AgCl gel EMG electrode. The strain-limiting 16-channel EMG e-skin had a high potential for daily applications, especially suitable for acting as a high-precision EMG acquisition module in daily rehabilitation training systems.

It is important to consider the physiological and psychological effects of the e-skin electrodes on users during long-term electrophysiological signal monitoring. To enhance the long-term wearing comfort of EMG e-skin electrodes and improve user experience, Miyamoto et al. proposed an inflammation-free and breathable e-skin based on nanomesh [45]. The nanomesh was made from water-soluble PVA nanofibers. A thin layer of Au was coated on the PVA nanofibers to form a dense conductive network (Fig. 2c). When the Au-PVA nanomesh was transferred onto human skin for EMG detecting, some water was sprayed on it to dissolve the PVA nanofibers and make the conductive nanomesh attach to human skin. Because the nanomesh e-skin was very thin and flexible and could be tightly fitted to the skin surface, the electrode–skin contact impedance was small enough (140 kΩ at 100 Hz) to monitor high-precision EMG signals without significant noise. Besides, the e-skin had high flexibility and durability and sustained over 10,000 cycles of bending test on a finger (with 40% elongation). Moreover, compared to other thin-film e-skins, the nanomesh e-skin was inflammation-free and not easy to cause skin irritation when worn for a long period. The nanomesh e-skin electrode could sustain the complex wearing environment in daily life and has huge potential in long-term daily EMG monitoring.

### Breathable EEG e-Skin Electrodes

EEG is an important electrophysiological signal which can reflect the condition of the human brain and is widely used in multiple health fields including fatigue detection, sleep condition monitoring, mental health diagnosis, etc. [46]. Nowadays, scalp electrodes are commonly used for detecting EEG signals [47]. However, EEG caps or rigid EEG electrodes are not suitable for daily long-term EEG monitoring because they are uncomfortable to wear and require complex preparation procedures. Therefore, to achieve daily long-term EEG signal monitoring, it is necessary to develop a more convenient wearing form on the one hand and a more comfortable electrode on the other hand [48]. At present, EEG signals have been able to be collected from places such as the forehead with high quality, eliminating the need to clean hair in advance before wearing electrodes on the scalp. To improve the wearing comfort and avoid sweat accumulation, the breathable EEG e-skin electrode is becoming an excellent choice for EEG monitoring on the bare skin. In this section, we will introduce some typical new breathable e-skin electrodes for daily long-term EEG monitoring.

The realization of a more comfortable and reliable EEG electrode has always been an important challenge to be solved in the application of long-term EEG monitoring in daily life. By using laser-scribed graphene and polyurethane (PU) nanomesh, Qiao et al. realized an EEG e-skin with high comfort, breathability, and high flexibility [5]. The PU nanomesh was fabricated by electrospinning PU-dimethyl formamide (DMF) solution. Graphene oxide (GO) nanoparticles were coated on PU fibers to form a core–shell PU-GO structure. The graphene oxide shell layer was further reduced by laser to form a conductive laser-scribed graphene (LSG) shell layer. The realized LSG-PU nanomesh e-skin was ultrathin and ultrasoft, when applied to the skin, the skin texture on the back could be clearly shown through the e-skin (Fig. 2d). The LSG-PU nanomesh e-skin had high flexibility (> 60% strain range), high stability (can sustain > 1000 stretching cycles), and excellent breathability (hardly prevents water vapor from being expelled). When choosing the conductive material of the e-skin, compared with noble metals like gold, the laser-scribed graphene was low cost, which made the EEG e-skin suitable for large-scale wear. The EEG signal acquired by the nanomesh e-skin had high quality, which could further be used as the input of the convolutional neural network to analyze the wearer’s concentration.

Personalized daily healthcare puts forward the requirement for low-cost, reliable, and comfortable medical equipment. 3D printing is a fast and customizable method for making breathable electrode templates. However, it remains a challenge to directly print highly flexible materials to achieve high-precision breathable structures. Facing this challenge, Ho et al. developed a 3D-printed sugar scaffold that was customized according to human characteristics and was used as a high-precision template for preparing flexible and porous EEG e-skins [49]. As shown in Fig. 2e, the human body shape characters were acquired by 3D scanning and used as the input of the 3D printer. The sugar grains formed a water-soluble 3D porous scaffold of the breathable e-skin by 3D printing. Then, the silicone elastomer was filled in the scaffold and the sugar scaffold was removed by water to form a silicone elastomer e-skin with a 3D porous structure. Finally, to give the e-skin conductivity, the surfaces of the porous silicone elastomer were coated with multiwalled carbon nanotubes to form a dense (4 kΩ/50 × 10 × 3 mm^3^) and light (0.25 g cm^−3^) conductive network. When detecting EEG signals, the customized porous e-skin contacted the skin surface with high conformability. The acquired EEG signals were used to effectively distinguish the sleep state of the wearer, which showed its capability of being used as a reliable, breathable, and personalized e-skin electrode.

Long-term wearable e-skin electrodes have the potential to be widely used in high-fidelity daily EEG signal monitoring. Ameri et al. developed a flexible and breathable graphene-tattoo-based EEG e-skin whose electrode–skin contact impedance is on par with the commercial Ag/AgCl gel electrode [13] (Fig. 2f). With a sub-micrometer thickness (~ 463 nm), the tattoo-like e-skin electrode has excellent electrical and mechanical properties. With over 40% strain range, the e-skin can be attached to human skin without restraining daily activities. More significantly, the tattoo electrode requires only one-tenth the area of the commercial Ag/AgCl gel EEG electrode to achieve the same contact impedance. Moreover, the e-skin collected EEG signals with high precision and clearly distinguished the eye-opening and eye-closing states without noise disturbing.

### Other Breathable e-Skin Electrodes

Apart from typical breathable e-skin electrodes mentioned above, there are also breathable e-skin electrodes developed for other applications. Although some of them are not designed for monitoring human physiological signals in daily life, the advanced technology and device performance adopted by them are also suitable for developing daily-use breathable electrophysiological signal e-skin electrodes. In this section, we will introduce two typical examples of the other breathable e-skin electrodes, the first is a breathable e-skin for detecting the health condition of plants, and the second is a breathable nanomesh e-skin for implantable ECG signal monitoring.

To realize physiological information monitoring of living fragile plants, Kim et al. invented a lossless way to directly vapor-printing conformal and durable electrodes on growing plants [50]. The p-doped poly(3, 4-propylenedioxythiophene) was chosen as the conductive material and applied to the surface of the plants by vapor-coating (Fig. 2g). The conductive polymer did not block or fill the stomata of the plant, and the vapor-printed electrode did not influence the natural growth of the plant specimens and could be used for a long period. This non-destructive method of preparing breathable electrodes without affecting normal physiological activities is likely to be transferred to the breathable e-skin electrode for monitoring human physiological signals in daily life, which shows its potential in the directly patterned breathable e-skin.

Implant or direct monitoring of physiological signals on tissues or cells requires higher breathability and biocompatibility of the e-skin, and its mechanical properties should meet the requirements that it will not cause damage to tissues or cells. Lee et al. used the Au-coated parylene-polyurethane nanomesh to monitor the field potential of the isolated cardiomyocytes [51] (Fig. 2h). The super-thin (only one or two layers of nanofibers) and ultra-flexible nanofibers did not hinder the motion of the cells. Besides, the nanomesh-based e-skin had ultra-low tensile force (0.4 mN) at a strain of 10%, which was much lower than the tension that cardiomyocytes can handle. With excellent biocompatibility, chemical stability, and ultra-high softness, the e-skin maintained a reliable operation for 96 h without performance degradation or damaging the cardiomyocytes, which showed its application potential in daily-use, no-body-damage breathable e-skin electrode.

In summary, we introduced some typical breathable e-skin electrodes for long-term electrophysiological signal monitoring in recent years and summarized their characteristics including types, breathability, materials, and fabrication methods. To facilitate parallel comparison, the characteristics of those typical breathable e-skin electrodes are listed in Table 1. Since most electrophysiological signal monitoring requires direct contact between the conductive material and human skin to record the signals with high quality, the design of the e-skin electrode should be considered to have a good fit with the skin to reduce the electrode–skin contact impedance while avoiding the noise interference signal caused by skin deformation and external environment. Besides, for daily long-term electrophysiological signal monitoring needs, electrode materials need good breathability, wear comfort, and stability. Moreover, considering the potential of daily large-scale use, the preparation process needs to be simplified and the cost can be reduced. To meet the above development requirements and ensure good breathability, the fiber-based or substrate-free e-skin electrode is becoming the preferred choice of breathable e-skin electrodes.

**Table 1:** Summary of the breathability, materials, and fabrication methods of the typical breathable e-skin electrodes listed in Sect. 2

Categories	Types	Breathability	Materials	Fabrication Methods	Refs.
ECG electrodes	Moisture-wicking and antibacterial e-skin	Water vapor transmission rate (WVT) ~ 70%	Dual-gradient poly (ionic liquid) nanofibers	Electrospinning	[29]
	3D conductive textile e-skin	20 g m^−2^ h^−1^	Conductive elastomeric melt-spun filaments	Industrial-scale knitting machine	[30]
	Substrate-free e-skin	hardly affects skin perspiration	Laser-scribed graphene	Sacrificial layer process	[31]
EOG electrodes	Tattoo-like e-skin	Breathable	CVD graphene	Sacrificial layer process	[36]
	Textile-based e-skin	Breathable	Graphene-coated commercial fabrics	Dip coating graphene oxide on fabrics and reducing	[19, 37]
	Soft-fabric-based e-skin	Breathable	20% silver and 80% polyamide, sponge package	Dip coated silver on the sponge	[38]
EMG electrodes	All-nanofiber-based e-skin	1748.09 g m^−2^ d^−1^	HPAN, PU, and AgNWs	Electrospinning and vacuum filtration	[43]
	Strain-limiting e-skin	WVT = 3.13 ± 0.18 g h^−1^ m^−2^	Gold electrode, polyimide supporting and packaging layer, porous elastomer-fabric substrate	E-beam evaporating and etching	[44]
	Inflammation-free e-skin	0.11 g day^−1^	Au-coated PVA nanofibers	Magnetron sputtering and sacrificial layer process	[45]
EEG electrodes	LSG-PU nanomesh e-skin	0.067 g day^−1^	Laser-scribed graphene, PU nanofibers	Electrospinning process and laser-scribing process	[5]
	3D-printed sugar scaffold template e-skin	Breathable	Silicone elastomer, SWCNTs	3D printing and sacrificial layer process	[49]
	Graphene-tattoo-based e-skin	Breathable	CVD graphene	Sacrificial layer process	[13]

## Breathable e-Skin Sensors

Driving by the increasing demand for daily long-term health monitoring, wearable sensors for monitoring various physiological signals in daily life have developed rapidly in recent years [52–55]. A variety of sensors have been developed to meet the various key physiological signal monitoring needs of healthcare. For the needs of daily long-term physiological signal monitoring, the design of breathable e-skin sensors has gradually become a research hotspot. Different breathable design methods have been proposed for the acquisition of different physiological signals, and a variety of advanced physiological signal monitoring methods have also been developed using well-designed breathable structures. In the following sections, we will introduce the recent development of breathable e-skin sensors according to the physiological signal types they monitored such as tactile, body motions, respiratory, pulse, skin surface humidity, body temperature, and glucose. Their design ideas, manufacturing methods, and performances will be discussed.

### Breathable e-Skins for Tactile Sensing

Tactile sensing is one of the human body’s most important abilities. In recent years, tactile sensors have played an important role in human–machine interaction, robotics, and medical health development [56]. To meet the needs of long-term physiological signal monitoring in daily life, a variety of tactile sensors with good breathability and high performances have been developed in recent years [14, 57–59]. In this section, we will introduce some recently developed representative breathable e-skins for tactile sensing.

**Fig. 3:**
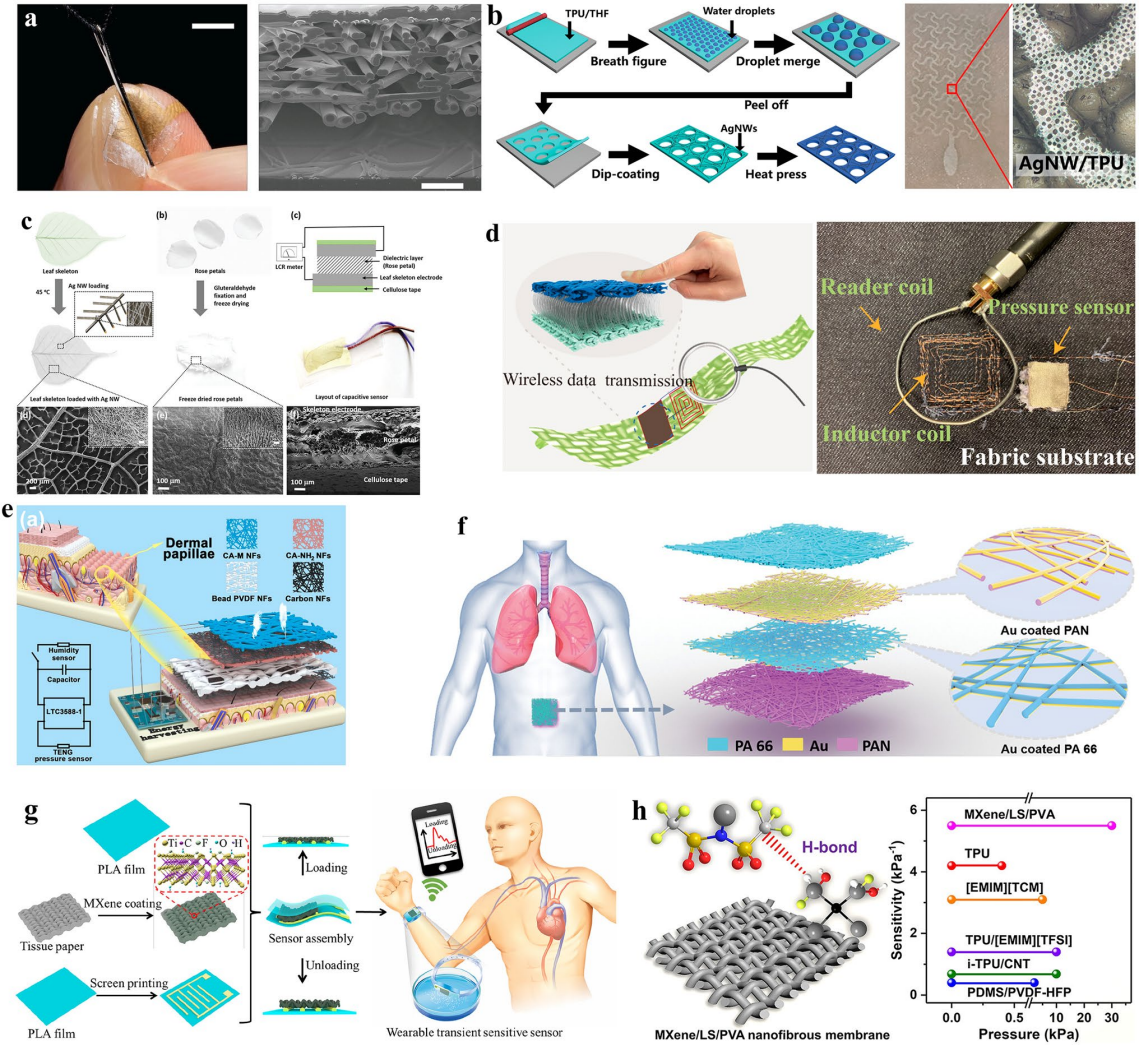
**a** Photograph and structure of the nanomesh-based tactile sensor. Reproduced with permission from Ref. [58]. Copyright 2020 AAAS. **b** The fabrication process of the porous TPU film and its photographs. Reproduced with permission from Ref. [59]. Copyright 2020 American Chemical Society. **c** The fabrication process and the structure of the plant-based body motion detecting e-skin. Reproduced with permission from Ref. [63]. Copyright 2020 Wiley. **d** Schematic illustration of the all-textile body motion detecting e-skin. Reproduced with permission from Ref. [64]. Copyright 2020 American Chemical Society. **e** The skin-inspired respiratory sensor. Reproduced with permission from Ref. [66]. Copyright 2022 Elsevier. **f** The structure of the all-nanofiber respiratory sensor. Reproduced with permission from Ref. [25]. Copyright 2021 Wiley. **g** The structure of the breathable and degradable pules sensor. Reproduced with permission from Ref. [67]. Copyright 2021 American Chemical Society. **h** The structure of the hydrogen-bond-triggered hybrid nanofibrous pulse sensor. Reproduced with permission from Ref. [68]. Copyright 2021 American Chemical Society

In human–computer interaction applications, if bulky wearable devices are used to record the human tactile, the actual recorded tactile signal is likely to be interfered with wearable devices and deviate from the real situation. To minimize the impact of tactile sensors on the actual feeling of the wearer, Lee et al. developed an ultrathin and ultrasoft breathable nanomesh-based tactile sensor and directly attached it to the fingertip to monitor the force felt by the fingertip [58]. As shown in Fig. 3a, the e-skin consisted of four layers including the Au top and bottom electrode layers, the polyurethane nanomesh layer, and the parylene coating layer. The breathable and ultrathin tactile sensor had little impact on the human’s natural sense of touch. Besides, the sensor showed high sensitivity to tactile pressure, with a 0.141 kPa^−1^ sensitivity under 1 kPa and a 0.010 kPa^−1^ sensitivity over 10 kPa. When grasping objects, the tactile sensor on the fingertip can precisely record the applied forces, which was of great significance in high-precision tactile perception and promising in human–computer interaction.

To meet the potential of large-area skin tactile sensing in daily life, the efficient preparation methods of breathable e-skin need to be realized. Zhou et al. developed a one-step fabrication method of the breathable tactile sensor based on a self-assembled porous substrate [59]. As shown in Fig. 3b, by a breath figure method, a porous thermoplastic polyurethane (TPU) substrate was prepared, and the conductive silver nanowires (AgNWs) were dip-coated on the substrate and was embedded into the porous substrate by a heat press process. This simple processing method greatly improved the preparation efficiency of breathable e-skins. When used as a breathable tactile sensor, it showed high water vapor permeability (23 mg cm^−2^ h^−1^), excellent stability, and low sheet resistance (7.3 Ω sq^−1^). Moreover, the porous TPU tactile sensor was further integrated into a wireless touch sensing system and realized a delicate tactile control of the software interfaces.

### Breathable e-Skins for Body Motion Detecting

Daily sports health management, sports medicine diagnosis, and medical rehabilitation training are inseparable from the accurate monitoring of human body motion. In daily life, as people have a higher pursuit of personal health, accurate recording and judgment of daily movements can help them achieve healthy and reasonable sports effects. In the medical field, if the patient’s motion can be quantitatively monitored, the accuracy and efficiency of medical diagnosis can be improved and the possibility of misdiagnosis can be reduced [21]. Facing the long-term body motion monitoring needs in daily life, and considering the wearing comfort and reliability comprehensively, many excellent breathable e-skins for body motion detection have been developed [60–64]. In this section, we will introduce some typical breathable motion-detecting e-skins for daily body motion monitoring.

As a breathable e-skin sensor for daily use, if it is low cost and degradable, it will be conducive to large-scale application in daily physiological signal monitoring. To create a biodegradable, disposable, and breathable e-skin for daily long-term body motion monitoring, Elsayes et al*.* developed a plant-based sustainable e-skin [63]. The fabrication process of the breathable body motion monitoring e-skin is shown in Fig. 3c. By using leaf skeletons loaded with AgNWs as the highly conductive top and bottom electrodes, and using the freeze-dried rose petals as the dielectric layer, a capacitive motion-detecting e-skin sensor was fabricated. Due to the structural characteristics of plants, the e-skin was naturally breathable based on the stomata in leaves and petals. At the same time, the fiber structure of plants endows the sensor with intrinsic flexibility and durability. The plant-based e-skin could maintain low resistance with 800 m^−1^ curvature without performance degradation after being pressed over 5000 times. When used to detect human body motions, the e-skin had a high sensitivity of about 0.08 kPa^−1^, which was suitable for high-precision body motion detecting. More significantly, the plant-based sensor was biodegradable, with the aid of water, the e-skin realized quick degradation in natural conditions, which shows its application prospect in sustainable wearable systems.

Daily long-term body motion-detecting requires the high durability of breathable e-skins. Combining functional materials with traditional clothing fibers, which have time-tested durability and biocompatibility, is a good solution to creating durable and skin-friendly e-skin. Wu et al. proposed an all-textile-based breathable e-skin for long-term human body motion monitoring [64]. The breathable e-skin was prepared by following steps. First, the PVA nanofibers were prepared by the electrospinning process and were used as the scaffold of the silver to form a conductive network. Second, the silver nanofibers were transferred to the top and bottom of a pre-stretched silk-based spacer fabric layer to form the highly durable and breathable capacitive sensor. The realized breathable e-skin has ultrahigh stability, which can sustain repetitive compression for more than 20,000 times, suitable for long-term body motion- detecting in daily life. Besides, with high sensitivity (0.283 kPa^−1^) and fast response time, the e-skin can realize high-precision dynamic motion detecting. Most importantly, the e-skin can be integrated into daily wear garments. As shown in Fig. 3d, with the aid of a conductive-fabric-knitted inductor coil, the motion signals recorded by the textile e-skin can be transferred to the mobile terminal wirelessly. The whole breathable textile-based motion detecting system can realize high-precision motion monitoring and signal transmission, which demonstrates its potential in daily exercise analysis and early warning of diseases.

### Breathable e-Skins for Respiratory Monitoring

Daily static or dynamic wearable respiratory monitoring is of great significance for disease prevention and health management [12]. In particular, sleep apnea syndrome which can greatly decrease people's quality of life, and the COVID-19 epidemic in recent years are all remind people to pay more attention to daily respiratory monitoring. Based on the social needs, in recent years, many excellent breathable e-skin respiratory monitoring sensors have been developed to meet the needs of daily long-term wear respiratory monitoring [25, 65, 66]. In this section, we will briefly introduce two typical breathable respiratory monitoring e-skins for daily long-term respiratory monitoring.

In long-term sleep respiration monitoring, the weak breathing signal has high requirements on the sensitivity of the respiratory sensors, and the usage scenarios in the sleep state have high requirements on the comfort and breathability of the sensor. By highly simulating the function of real skin in structure, Yue et al. realized a self-powered, low-cost, and highly sensitive respiratory sensor by electrostatic spinning [66]. As shown in Fig. 3e, by using the bead polyvinylidene fluoride (PVDF) nanofibers to simulate the dermal papillae of human skin and combining the collagen aggregate nanofibers, the realized sensor shows high sensitivity (0.32 V kPa^−1^). The breathable skin-inspired respiratory sensor can record the breathing state during sleep clearly, and it is also biocompatible with the skin and does not cause discomfort to the wearer because of its light-weight, softness, and good breathability. Besides, the respiratory sensor can also be used to monitor humidity and pulse, which can be combined with the respiratory monitoring function to form a comprehensive evaluation system of human sleep quality.

Apart from the sleep state, in daily respiratory monitoring, if the frequency, intensity, and interval of respiration can be monitored comprehensively without feeling, it is of great significance for health management and disease diagnosis. Such imperceptible sensors can be implemented in an all-nanofiber fashion. Peng et al. used the multilayer polyacrylonitrile and polyamide 66 nanofibers to fabricate an all-nanofiber breathable respiratory sensor [25]. As shown in Fig. 3f, the sensor consists of four layers of nanofibers including the top encapsulation polyamide 66 layer, the electrification layers consist of the Au coated polyacrylonitrile layer, the Au coated polyamide 66 layer and the bottom polyacrylonitrile substrate layer. The acquired breathable, flexible, and self-powered e-skin can monitor the respiratory signal of the human in real-time with detailed respiration frequency, intensity, and interval information. They also used the e-skin to comprehensively evaluate the obstructive sleep apnea–hypopnea syndrome (OSAHS), which shows the clinical diagnostic value of the high-precision signals it can be recorded.

### Breathable e-Skins for Pulse Sensing

Pulse is one of the most important physiological signals of the human body, which can reflect the basic state of human health [67]. People’s attention to cardiovascular disease has also led to the birth of many wearable pulse monitoring devices, among which, the development of breathable e-skin pulse sensors suitable for daily long-term wear is particularly prominent in recent years [68]. In this section, we will introduce two typical breathable pulse sensors and explain why they are suitable for daily pulse monitoring.

With the extensive use of wearable electronics, a lot of e-waste is generated, which has gradually developed into a serious environmental problem. If low-cost, daily-use wearable devices can achieve good functions while easy to degrade, they will be competent for large-scale applications in the consumer market. For developing a wearable pulse monitoring sensor suitable for daily use, Guo et al. proposed an easily degradable and low-cost breathable pulse sensor [67]. As shown in Fig. 3g, the sensor was made of breathable and recyclable tissue paper coated with MXene and encapsulated with biodegradable PLA films. The heart beats an average of 100,000 times in a day, and the signals are weak on the skin surface, the acquired sensor should be highly sensitive (with a 10.2 Pa low detection limit) and durable (can sustain over 10,000 cycles of press). It is worth noting that the e-skin sensor can be degraded in the NaOH solution in two weeks, which shows its potential in daily large-scale applications.

On the surface of the human body, the pulse is a tiny physiological signal, which requires the high sensitivity of the sensor. Meanwhile, the interference of human activities in daily life raised higher requirements for the signal monitoring range of the pulse sensor. However, it has always been a challenging research topic to develop flexible and breathable sensors with high sensitivity and a large measuring range. Sharma et al. used a hydrogen-bond-triggered hybrid nanofibrous membrane to solve this problem [68]. As shown in Fig. 3h, the fabric sensing material consists of a polymer electrolyte made of poly (vinyl alcohol) and the superhydrophilic bis(trifluoromethane) sulfonamide lithium salt. The Ti_3_C_2_T_x_ MXene is introduced into the polymer electrolyte to realize an ion confinement effect, which helps the fabricated breathable pulse sensor obtain high sensitivities of 5.5 kPa^−1^ in the 0 ~ 30 kPa measuring range and 1.5 kPa^−1^ in the 30 ~ 250 kPa measuring range. When used to detect the pulse signal of a human, the sensor can clearly distinguish dicrotic peaks, main peaks, and predicrotic peaks from the waveform. Based on these parameters, related cardiovascular diseases can be diagnosed.

### Breathable e-Skins for Skin Surface Humidity Sensing

The skin surface humidity is of great significance to human health, which can be used to judge the development of skin diseases and assess the metabolic level of humans. Current e-skin sensors are ideal for monitoring epidermal moisture because they fit tightly with the skin. However, to avoid the e-skin affecting the normal moisture changes of the skin, its breathability is essential. In this section, we will introduce the recent excellent work in the field of the breathable skin surface humidity e-skin, learning the preparation processes of their breathable structures, device performances, and applications.

**Fig. 4:**
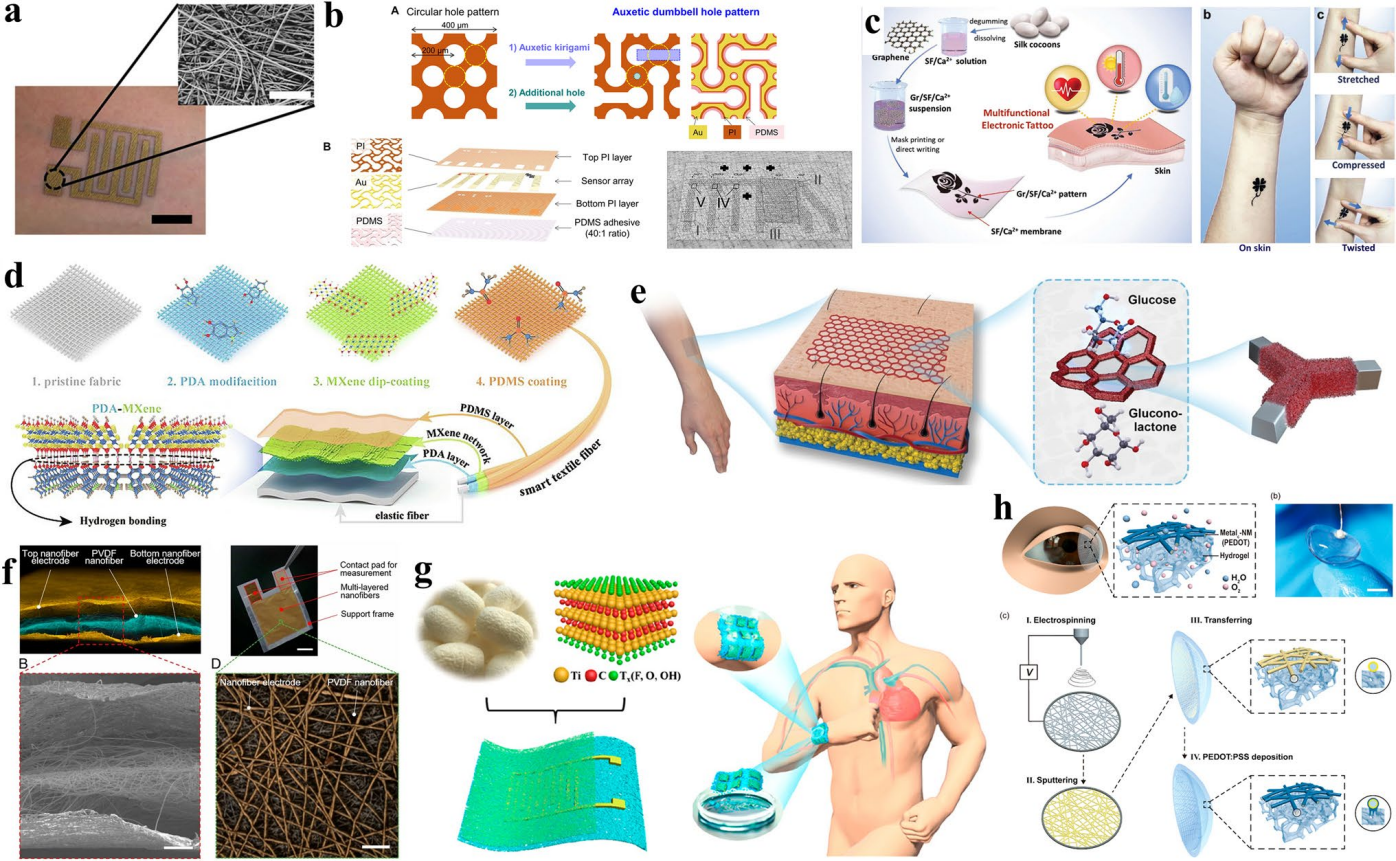
**a** Photograph and SEM image of the nanomesh-based humidity sensor. Reproduced with permission from Ref. [69]. Copyright 2020 Wiley. **b** Structure of the breathable e-skin inspired by the sweat pore. Reproduced with permission from Ref. [70]. Copyright 2021 AAAS. **c** The fabrication process of the self-healable electronic tattoos. Reproduced with permission from Ref. [74]. Copyright 2021 Wiley. **d** Preparation process of the superhydrophobic textile-based e-skin. Reproduced with permission from Ref. [75]. Copyright 2021 Elsevier. **e** The breathable and self-supporting glucose sensor. Reproduced with permission from Ref. [76]. Copyright 2021 Wiley. **f** The structure of the all-nanofiber-based cardiac sound sensor. Reproduced with permission from Ref. [77]. Copyright 2020 PANS. **g** Schematic illustration of the silk-MXene human voice detector. Reproduced with permission from Ref. [78]. Copyright 2021 American Chemical Society. **h** The structure of the breathable contact lens. Reproduced with permission from Ref. [79]. Copyright 2019 American Chemical Society

To make the skin surface humidity condition as close as possible to the bare skin condition, the e-skin needs to be breathable and as imperceptible as possible. Matsukawa et al. used a nanomesh-based e-skin to record the skin impedance and acquire the skin surface humidity [69] (Fig. 4a). With porous PVA nanomesh substrate coated with a gold conductive layer, the e-skin achieved high water vapor permeability and high conductivity. Since the nanomesh was soft, biocompatible, and flexible when attached to the human skin, the wearer did not feel the existence of the e-skin, and no allergy or redness was discovered during the long-term wearing. Compared to the rigid humidity detecting probes used clinically, the flexible nanomesh-based e-skin caused no significant pressure on the skin surface to disturb the normal skin environment. In addition, the breathable structure of the e-skin sensor ensured that the skin could normally emit moisture so that the measured humidity value was accurate. By using the nanomesh-based e-skin to continuously record the changes in the human skin surface humidity, the water loss of the cuticle can be assessed, and some typical skin diseases can be preliminarily diagnosed based on the acquired skin surface humidity information.

To ensure reliable skin surface humidity measurements, the interface environment between human skin and e-skin should be stable. The human body can ensure the relative stability of the internal environment through a variety of self-regulation mechanisms. Among many tissues and organs used to regulate the balance of the human internal environment. Yeon et al. inspired by the sweat pore, proposed a perforated e-skin that can efficiently assist in the excretion of sweat from the sensor-skin interface [70]. As shown in Fig. 4b, the layout of the e-skin is similar to the art of paper-cut. The holes on it allow the sweat to spread out and help to maintain the normal physiological activities of the skin. Between the holes, some exquisitely designed kirigami patterns were made to increase the flexibility of the e-skin and ensure the wearing’s comfortability. Besides, this breathable structure required a delicate sensor components layout to ensure the smooth realization of humidity detection function. The acquired e-skin showed high moisture transmittance up to 94.54% (± 7.33%), indicating that the e-skin could perform its function even when the wearer sweating a lot. Besides, a long-term skin surface humidity monitoring experiment (over one week) was successfully carried out without disturbing the daily activities of the wearer. With good mechanical reliability, conformability, and high detecting performance, the sweat pore-inspired breathable e-skin is promising for long-term skin surface humidity monitoring in daily life.

### Breathable e-Skins for Body Temperature Sensing

Body temperature is an important indicator of our health conditions, and it is also an important indicator of the disease or wound progression [71]. Body temperature is a physiological parameter that changes continuously and dynamically. Compared with a single measurement, continuous daily body temperature monitoring has more important clinical significance [72]. Temperature monitoring e-skins are suitable for long-term continuous body temperature monitoring, which has developed rapidly in recent years [73–75]. Since the range of body temperature is not large even under different circumstances, the monitoring accuracy of the sensor is important. To avoid the influence of airtight e-skin on body temperature measurement accuracy (for example, the poor thermal conductivity of e-skin substrate may lead to a higher temperature measurement result), the breathable body temperature e-skin with good thermal conductivity has attracted much attention these years [74, 75]. In this section, we will introduce some typical breathable e-skins for high-precision body temperature sensing.

Most breathable e-skins have the characteristics of lightweight, thin, and soft. Although these characteristics can make the wearer more comfortable and e-skins suitable for daily long-term wear, they inevitably affect their durability and stability. Instead of cutting costs and replacing devices frequently, it is a good idea to extend the life of devices by giving them self-healing capabilities. Based on silk and graphene, Wang et al. realized a self-healable breathable e-skin for long-term body temperature monitoring [74]. The fabrication process is shown in Fig. 4c, natural silk was dissolved to obtain the silk fibroin/Ca^2+^ solution, and the graphene nanoplates were added to the solution to make a graphene/silk fibroin/Ca^2+^ suspension. The suspension was directly written or printed on the silk fibroin/Ca^2+^ substrate, and the acquired tattoo-like e-skin can be transferred to human skin only with the help of water. The self-healing mechanism of the e-skin comes from the swollen silk fibroin/Ca^2+^ matrix and the reformation of bonds. The broken e-skin could be reconnected only with the help of a small amount of water, which significantly improves its durability in long-term wearing. The temperature-sensing ability comes from the electron hopping between the graphene nanoplates. When used to monitor the human body temperature, the e-skin shows high precision, high response speed, and high stability in a 20 ~ 50 °C large detecting range, which shows its capability in daily long-term body temperature monitoring.

Due to their unique application environments, e-skins may face long-term sweat erosion and complex environmental damage. This puts forward a high demand for their stability. In addition, to avoid affecting the breathability of the e-skin, it is necessary to design a breathable package so that it not only does not affect the temperature signal monitoring but also protects the device to the maximum extent. Luo et al. proposed a superhydrophobic and breathable textile-based e-skin for body temperature sensing [75]. The preparation process of the e-skin is shown in Fig. 4d. First, the elastic textile was modified by polydopamine. Second, the MXene nanoplates were coated on the surface of the polydopamine with the help of plenty of functional groups on the MXene nanoplates that can absorb tightly on the polydopamine layer. Third, a PDMS layer was used to package the e-skin to avoid the MXene being oxidized or destroyed while forming a superhydrophobic layer. With high hydrophobic and high breathability, the e-skin was easy to clean and comfortable to wear. With the excellent photo-electro-thermal response, the breathable e-skin showed a wide temperature measuring range, high sensitivity, and high repeatability, meaning that it was competitive in smart wearable body temperature monitoring devices.

### Other Breathable e-Skin Sensors

In addition to the breathable e-skin for the daily monitoring of several physiological parameters listed above, there are also ingeniously designed breathable e-skin sensors for the daily monitoring of many other physiological signals such as blood pressure, glucose, cardiac sound, and human voice [55, 76–79]. In this section, we will introduce several typical breathable e-skin sensors for other physiological information acquisition in daily life.

Continuous monitoring of blood pressure in daily life is important for analyzing a variety of cardiac health conditions. Realizing non-obtrusive blood pressure monitoring is still a meaningful and challenging task. To achieve high precision, non-obtrusive daily continuous blood pressure monitoring, Kireev et al. proposed a breathable graphene bioimpedance tattoo-like e-skin [55]. CVD graphene was transferred onto the human skin with the help of PMMA and tattoo paper. The tattoo-like e-skin can realize intimate location-stable contact with skin, which ensures that the dynamic impedance can be measured stably to calculate blood pressure while ensuring breathability and comfort. The e-skin can achieve an accuracy that exceeds previous reports with 0.06 ± 2.5 mmHg (diastolic blood pressure) and 0.2 ± 3.6 mmHg (systolic blood pressure). Besides, the e-skin does not interfere with patients during daily wear, suitable for continuous blood pressure monitoring.

Glucose concentration is an important physiological parameter for the daily healthy diet and the management of diabetes. Compared to invasive glucose monitoring methods, epidermal glucose monitoring with the e-skin is more suitable for daily use. Li et al. proposed a skin-like, micromesh-based e-skin for glucose monitoring [76]. As shown in Fig. 4e, first, the self-supporting Ni micromesh was fabricated by direct laser writing and selective electrodeposition to form a breathable scaffold for the catalyst. Then, Cu_2_O was grown on the micromesh by electrochemical deposition. With a self-supporting structure, the e-skin shows high conformability to the skin surface. Here is the working mechanism of the e-skin: the sweat glucose is converted to gluconolactone by Cu_2_O, resulting in a current change according to the glucose concentration. The current change can be measured by the e-skin. During the real-time glucose concentration monitoring, the e-skin shows high sensitivity (15,420 µA cm^−2^ mM^−1^), low-detecting limit (50 nM), and fast response time (< 2 s), which shows the breathable glucose sensor is qualified for the daily glucose monitoring.

Continuous monitoring of cardiac sound is crucial for the prevention and diagnosis of cardiovascular diseases. Cardiac sound is characterized by low frequency and low intensity, and its long-term monitoring requires high sensitivity and high comfort for breathable e-skins. Nayeem et al. developed an all-nanofiber-based breathable mechano-acoustic sensor for ultrasensitive long-term cardiac sound monitoring [77]. The sensor consists of three layers of nanofibers (Fig. 4f). The top and bottom electrode layers were made of Au-coated polyurethane nanomesh, and a PVDF nanomesh was used as the piezoelectric layer. The all-nanofiber-based structure shows excellent breathability, which can be reflected by its 12.4 kg m^−2^ d^−1^ water vapor permeability. With ultra-high sensitivity (10,050.6 mV Pa^−1^) in the low-frequency range (< 500 Hz), the e-skin realized long-term (10 h) monitoring of the cardiac sound with a high signal-to-noise ratio (SNR) (40.9 dB), which shows its potential in daily cardiovascular health monitoring.

Speaking is an important way for people to interact with the outside world. For some disabled people, even though they can’t vocalize, their larynxes can vibrate, which may be further converted into meaningful sound messages. The human voice sensor is a vital tool to accomplish this transformation process. Since people often interact with the outside world in daily life, the voice sensor needs to be comfortable to wear and breathable. Chao et al. realized a breathable MXene-protein e-skin for ultrasensitive human voice detection [78]. The schematic illustration of the breathable e-skin is shown in Fig. 4g. The porous substrate of the e-skin was made from natural silk obtained from the silkworm by electrospinning. The Ti_3_C_2_T_x_ MXene nanoplates were attached to the silk substrate through supramolecular interactions and formed the MXene-silk sensing layer. The electrode layer was made of silk substrate with MXene-ink printed electrode pattern. After the face-to-face assembly, the two layers were integrated and formed a breathable e-skin for human voice monitoring. The sensor presented ultrahigh sensitivity in a wide range (0 ~ 39.3 kPa) and good mechanical durability (remains stable after 10,000 cycles of loading–unloading tests), which was promising in daily human voice detecting.

Contact lenses, which are in direct contact with eyeballs and tears, are a very promising e-skin platform that can be endowed with many functions for daily health monitoring. However, the human eye is a relatively fragile part, which has high requirements for the irritation-free design, the biocompatible material, and the breathable structures of contact lenses. Wei et al. proposed a breathable and irritation-free contact lens sensor based on metal-coated nanomesh to realize long-term electroretinogram recording during daily wear [79]. A thin-film nanomesh prepared by the electrospinning polyacrylonitrile (PAN) was used to form a transparent and breathable substrate suitable for prolonged contact with the eyeball. After being coated by an Au layer, the Au-PAN nanomesh shows ~ 51.23 Ω sq^−1^ resistance at 95.1% transmittance. To increase its adhesion to the hydrated hydrogel, a layer of PEDOT:PSS was coated on the Au-PAN nanomesh by the electrochemical deposition to form a connector between the nanomesh and the hydrogel (Fig. 4h). The acquired breathable contact lens shows high breathability, good wettability, and biocompatibility. When used to monitor the electroretinogram signals in a rabbit’s eye, the contact lens successfully recorded the signals of the rabbit without causing abnormalities in its eye. The breathable contact lens sensor can be used to diagnose retinopathy and can serve as a good platform for carrying more physiological sensors. Besides, it is suitable for daily use and it can place a less psychological burden on the wearer than traditional ophthalmic devices.

In summary, taking typical epidermal physiological signals as examples, the development of breathable e-skin sensors in recent years is introduced. Based on the sensing requirements of different physiological signals, we summarized their characteristics including sensor types, breathability, materials, and fabrication methods. To facilitate parallel comparison, the characteristics of those typical breathable e-skin sensors are listed in Table 2. Because the suitable monitoring sites of different physiological signals are different, the monitoring methods of the same physiological signal may also different, and the breathability, materials, and fabrication methods for specific sensors need to be individually designed. Besides, for daily long-term physiological signal monitoring, like the e-skin electrode, the sensing materials need good breathability, wear comfort, and stability. To meet the above development requirements and ensure good breathability, Electrospinning is becoming the preferred choice of breathable e-skin sensors.

**Table 2:** Summary of the breathability, materials, and fabrication methods of the typical breathable e-skin sensors listed in Sect. 3

Categories	Types	Breathability	Materials	Fabrication Methods	Refs.
Tactile sensors	Nanomesh-based tactile e-skin	Breathable	Au, PU nanomesh, parylene coating	Electrospinning	[58]
	Self-assembled porous e-skin	23 mg cm^−2^ h^−1^	Porous thermoplastic TPU, AgNWs	Dip-coating, heat pressing	[59]
Body motion sensors	Plant-based sustainable e-skin	Breathable	Leaf skeletons, AgNWs, freeze-dried rose petals	Freeze-drying	[63]
	All-textile-based e-skin	Breathable	PVA nanofibers, silver nanofibers	Electrospinning	[64]
Respiratory sensors	Dermal papillae simulated e-skin	18.5 mm s^−1^	Bead PVDF nanofibers	Electrospinning	[66]
	All-nanofiber respiratory e-skin	~ 10 mm s^−1^	Polyamide 66 nanofibers, Au, polyacrylonitrile	Electrospinning	[25]
Pulse sensors	Degradable pulse e-skin	Breathable	Recyclable tissue paper coated with MXene and encapsulated with PLA films	Dip-coating, screen printing	[67]
	Hydrogen-bond-triggered hybrid nanofibrous e-skin	Breathable	Polymer electrolyte made of PVA and the superhydrophilic bis (trifluoromethane) sulfonamide lithium salt, MXene	Electrospinning	[68]
Skin surface humidity sensors	Nanomesh-based e-skin	1.9 mg cm^−2^ h^−1^	PVA nanomesh, gold conductive layer	Electrospinning	[69]
	Perforated e-skin	VTR = 94.54% (± 7.33%)	PI, Au, PDMS, etc	Photoresist-based lift-off process, e-beam evaporation, spin coating, etc	[70]
Body temperature sensors	Self-healable e-skin	Breathable	Silk and graphene	Directly writing/printing silk fibroin/Ca^2+^/graphene solution	[74]
	Superhydrophobic textile-based e-skin	0.55 kg m^−2^ h^−1^	MXene nanoplates, polydopamine substrate, PDMS package	Dip-coating	[75]
Blood pressure	Graphene tattoo-based e-skin	Breathable	CVD graphene	Sacrificial layer process	[55]
Glucose sensor	Micromesh-based e-skin	> 2500 mm s^−1^ at 10 Pa	Ni micromesh, Cu_2_O	Direct laser writing, selective electrodeposition, electrochemical deposition	[76]
Cardiac sound sensor	All-nanofiber-based mechano-acoustic e-skin	12.4 kg m^−2^ d^−1^	Au-coated PU nanomesh, PVDF nanomesh	Electrospinning	[77]
Human voice sensor	MXene-protein e-skin	~ 0.05 g day^−1^	MXene nanoplates, silk substrate	Ink printing, face-to-face assembly	[78]
Electroretinogram recording sensor	Irritation-free contact lens e-skin	~ 46.2 mg cm^−2^ day^−1^	Au-PAN nanomesh, PEDOT:PSS	Electrospinning, electrochemical deposition	[79]

## Breathable e-Skin Systems

Breathable e-skin systems for daily health monitoring are becoming a research hotspot these years. To become more suitable for long-term use in daily life, breathable e-skins have gradually developed from the device level that can only detect physiological signals to the system level. This trend has led to several important changes: (1) The breathable e-skin can detect more kinds of physiological signals by integrating a variety of breathable sensors and electrodes [22, 80–85]. (2) The breathable e-skins are becoming more integrated. Apart from the basic physiological signal monitoring function, they are endowed with signal storage, signal processing, signal transmission, disease treatment, and other functions [71, 86–90], which are suitable for daily independent work to realize the in-situ sensing-processing-diagnosis capability. (3) e-Skins are getting smarter. They can use intelligent algorithms to classify or diagnose physiological signals to assess health conditions and even diagnose diseases [5, 31, 91–93]. In the following sections, we will introduce the breathable e-skin systems developed with these three changes and show their advantages in daily healthcare.

### Multifunctional Breathable e-Skin Systems

Human health assessment and disease diagnosis are usually not based on a single physiological signal, but on a combination of multiple physiological signals to conclude. Facing the need of monitoring a variety of physiological signals of the human body and the demand of performing other functions, the breathable e-skins are becoming multifunctional [22, 75, 80–84]. In this section, taking the typical breathable e-skin systems developed in recent years as examples, we will discuss the multifunctional challenges faced by the breathable e-skin and corresponding solutions, and introduce design ideas and characteristics of multifunctional breathable e-skin systems.

**Fig. 5:**
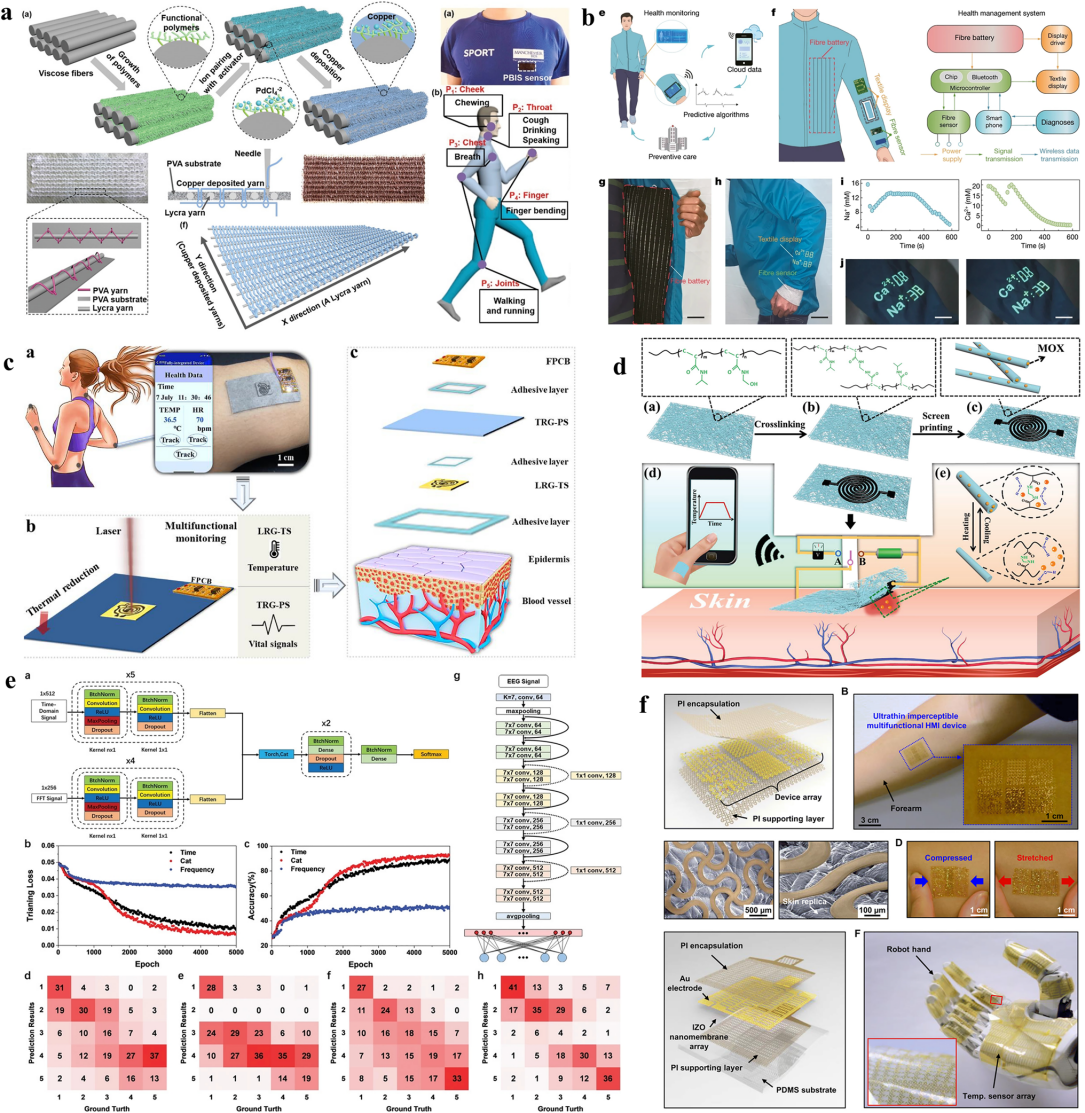
**a** Schematic illustration of the multifunctional and anti-jamming e-skin system and its preparation method. Reproduced with permission from Ref. [83]. Copyright 2021 Springer Nature. **b** A versatile breathable textile-based e-skin system. Reproduced with permission from Ref. [84]. Copyright 2021 Elsevier. **c** The fully integrated breathable e-skin system based on graphene-cellulose paper. Reproduced with permission from Ref. [89]. Copyright 2021 Elsevier. **d** The breathable e-skin system with temperature sensing and wound treating abilities. Reproduced with permission from Ref. [72]. Copyright 2019 Wiley. **e** The intelligent algorithm model used by the graphene nanomesh e-skin. Reproduced with permission from Ref. [5]. Copyright 2022 Wiley. **f** The breathable e-skin used for human–machine interfaces. Reproduced with permission from Ref. [93]. Copyright 2019 AAAS

To realize the monitoring of various physiological signals, the breathable e-skin system has been endowed with more functions. On the one hand, this requires that the sensitivity and range of sensors on the e-skin can meet the monitoring needs of various physiological signals. On the other hand, in addition to the range of measurements, the e-skin needs to be sensitive only to desired physiological signals and insensitive to irrelevant ones. To realize an all-in-one breathable e-skin with anti-jamming capability, Liu et al. proposed a multifunctional e-skin system based on strain sensing which is insensitive to pressure and bending [83]. The e-skin system was prepared through the following steps: First, the conductive fibers were proposed by growing copper on viscose fibers through a polymer-assisted metal deposition method (Fig. 5a). Second, the conductive fibers were woven into breathable strain sensors by an embroidery machine. The device performance can be flexibly adjusted by the design of the fiber size and the weaving method. The acquired e-skin system shows high sensitivity and a large measuring range to strain (with a gauge factor up to 49.5 in 0 ~ 100%). More significantly, it can neglect pressure and bending noise signals. As shown in Fig. 5a, the breathable e-skin could detect a variety of strain-based physiological signals including chewing, speaking, breath, finger bending, walking, etc. This multifunctional, anti-jamming, and breathable e-skin system is ideal for detecting physiological signals in daily life with complex environmental interferences.

In addition to monitoring multiple physiological signals, the breathable e-skin can be integrated with a variety of functions including display and information interaction to realize a daily physiological signal monitoring system. Based on their ingenious large-scale multifunctional textile [80], He et al. realized a versatile textile-based breathable e-skin system [84]. As shown in Fig. 5b, by integrating the fiber sensor, the textile display, the fiber battery, and the microprocessor, the realized textile-based breathable e-skin can realize a whole physiological signal monitoring-processing-diagnosis function. The real-time Na^+^ and Ca^2+^ concentration in human sweat can be recorded and displayed on the textile e-skin, and the physiological information can be transferred to mobile devices or hospitals for further processing. Besides, the entire system can be integrated with garments easily and its applications can be extended to processing other physiological signals, meaning that it is suitable for daily long-term multiple physiological signal monitoring. The textile-based breathable e-skin system is promising in personal health management in daily life and is especially useful for the early diagnosis of diseases.

### Integrated Breathable e-Skin Systems

The integration of breathable e-skin is becoming a developing trend in recent years. During the long-term daily wearing process, if the collected physiological signals can be processed in situ, or if the e-skin system can realize in situ diagnosis and treatment, the wearer will get great convenience. In fact, in addition to the basic physiological signal monitoring function, more and more breathable e-skin systems are endowed with signal processing, transmission, diagnosis, treatment, self-supply, or other functions, and the degree of integration is greatly improved [72, 86–89]. In this section, we will introduce two typical full-integrated breathable e-skin systems and demonstrate the advantages of improved integration for breathable e-skins.

Zhang et al. realized a fully integrated breathable e-skin system that could be used in long-term monitoring and transferring of the body temperature and motions [89]. Based on paper made from cellulose microfibers combined with graphene oxide, the realized graphene-cellulose paper acquired good washability and breathability. The temperature sensor and pressure sensor were made on the same piece of graphene-cellulose paper by the laser-scribing method and the thermal reduction method, respectively. To realize a fully integrated system with long-term signal sensing and transferring abilities, as shown in Fig. 5c, they integrated the two sensors, an FPCB, and a microcontroller to form a breathable e-skin system with physiological signal sensing, processing, and transferring abilities. The acquired temperature and motion signals can be further transferred wirelessly to a smartphone for display. With high breathability and washability, the e-skin system can be worn for at least 7 days without causing discomfort to the wearer or blocking the release of sebum and sweat, while the system itself maintains good performance. The fully integrated breathable e-skin system may create a new era of long-term, daily-use healthcare electronics.

If the breathable e-skin system can not only monitor and diagnose physiological signals but also has disease treating ability, the constructed doctor-like system is of great significance for the rehabilitation of disease in daily life and can alleviate the shortage of medical resources. Gong et al. developed a breathable e-skin system with temperature sensing and wound infection treatment capabilities [72]. As shown in Fig. 5d, the temperature sensing module of the e-skin was prepared by screen printing silver ink on the breathable substrate. The substrate was made from cross-linked electrospinning moxifloxacin-hydrochloride-loaded poly (N-isopropyl acrylamide-co–N-methylol acrylamide) nanomesh with thermo-responsive. The breathable e-skin system was attached to the wound to monitor temperature changes in real-time and wirelessly transmits the information to a smartphone. The working principle of the e-skin system is as follows: The e-skin system is attached to the wound, and the temperature sensing module on it can monitor the body temperature continuously and transmit it wirelessly to a smartphone in real-time. Once the temperature sensing module detects an abnormal temperature change (which indicates that the wound is infected), the control module of the e-skin system makes the heating module heat the thermo-responsive electrospinning substrate to release the moxifloxacin hydrochloride, which can eliminate the wound infection. In particular, the breathable and biocompatible e-skin is particularly suited for use at the wound site, and its heating power facilitates wound healing in addition to aiding drug release. Besides, temperature modules on the e-skin can be used for heating and sensing temperature at the same time, facilitating the realization of a multifunctional system. This doctor-like system has both physiological signal diagnosis and wound treatment capabilities, which shows its significant development prospects in patient monitoring, human–computer interaction, and other fields.

### Intelligent Breathable e-Skin Systems

The development of artificial intelligence provides a new opportunity for e-skin systems to be widely used in daily life. Traditional e-skin systems may have the ability of physiological signal monitoring, storage, and transmission, but most of them have shortcomings in signal processing and feedback. If an e-skin system cannot interact with the wearer and analyze the collected signals efficiently, it may hinder its large-scale application. Facing these problems, many researchers are trying to intelligently process the physiological signals collected by the e-skin to improve the physiological signal processing efficiency and assist doctors in disease diagnosis, or try to enable e-skin systems to autonomously give feedback to the wearer [5, 31, 90–93]. In this section, we will introduce typical research on the breathable e-skin system with intelligent diagnosis function and the e-skin system with intelligent feedback capability.

The analysis of human attention through the EEG signal is of great significance for driving safety-enhancing, fatigue monitoring, and psychological state assessment. To improve the efficiency of signal analysis, Qiao et al. combined the breathable graphene nanomesh e-skin and the intelligent algorithm for EEG analysis to form an intelligent breathable EEG system [5]. By using the breathable graphene nanomesh mentioned in Sect. 2.4 as the EEG electrode to collect the high-quality EEG signal [31], and using a multilayer convolutional neural network (CNN) to analyze the EEG signals intelligently. As shown in Fig. 5e, since the EEG signal has low intensity and contains both time domain and frequency domain information, a concatenated CNN model was applied to improve the accuracy of the EEG diagnosis. By dividing people's attention into five levels and intelligently classified EEG signals recorded by breathable e-skin electrodes, the classification accuracy of the intelligent breathable e-skin system can reach 99% for training sets and 48.7% for test sets. This intelligent breathable e-skin system may shed light on the “doctor-like” disease early warning system and the daily-use mental health monitoring systems.

In Sect. 4.2, we have mentioned integrated breathable systems that can transfer the physiological signal wirelessly to a smartphone for waveform display or further processing [72, 89]. However, if there is a breathable e-skin system that can interact with humans and direct feedback analysis results of the acquired physiological information, such a closed-loop system would greatly improve the flexibility of use. Sim et al. used the metal oxide semiconductor nanomembrane to form an imperceptible and breathable closed-loop e-skin system [93]. As shown in Fig. 5f, the breathable e-skin system consists of ReRAM and FET arrays, distributed temperature sensors, UV sensors, strain sensors, and thermal stimulators. All devices are flexible and were prepared on a breathable IZO nanomembrane and connected by the serpentine Au electrodes. The system could monitor physiological signals including body temperature, motions, and UV light which had a great impact on human health. Most importantly, it demonstrated outstanding capabilities in human–machine interaction. By detecting and analyzing human motions, the e-skin system could control a robotic hand with high sensitivity. Besides, if the e-skin system was attached to a prosthesis to touch objects or people with different temperatures, the feeling of temperature “perceived” by the prosthesis will be given to the human by the thermal stimulator in the breathable e-skin attached to the human skin. This closed-loop smart e-skin system is not only suitable for daily wear because of its breathability and imperceptibility but can also realize timely feedback of acquired information, which shows its promising prospects in human–machine interfaces for healthcare.

In summary, in view of the systematic development trend of breathable e-skin, new types of breathable e-skin systems with multifunction, integration, or intelligence for daily health monitoring are introduced with examples. The systematic development trend of breathable e-skin enables e-skin to have more physiological signal monitoring capabilities and integrate more functions, while the e-skin system may collect, process, and transmit signals in situ and even realize intelligent diagnosis or act as human–computer interaction, which adapts to the needs of daily long-term use of e-skin. However, our classification of systems into three categories does not mean that each e-skin system has only a specific set of characteristics. In recent years, many excellent reports on breathable e-skin systems have made breathable e-skins combine two or three of these characteristics including multifunctional, integrated, and intelligent, not just one. In addition, the systematic trend brings new problems to be solved for breathable e-skin, including: (1) how to ensure the breathability of e-skin with a higher density of functions or modules and (2) how to choose the appropriate wearing position and system layout under the condition of higher integration, so as to ensure the simultaneous high-quality collection of various physiological signals, etc. In the next section, we will discuss some aspects of the development of breathable e-skin for physiological signal monitoring.

## Discussion and Outlook

In the above sections, we systematically introduce breathable e-skins for daily physiological signal monitoring developed in recent years. By dividing them into breathable e-skin electrodes, breathable e-skin sensors, and breathable e-skin systems, we introduce them in detail, sort out their design ideas, manufacturing processes, performances, and applications and show their advantages in long-term physiological signal monitoring, respectively.

In the above sections, the characteristics of breathable e-skins in recent years are summarized through a parallel comparison of different physiological signal monitoring types, breathabilities, materials, and fabrication methods of some typical examples. In the fabrication methods and materials, an outstanding feature is that electrospinning fabrics based on organic materials play an important role in breathable e-skin electrodes and sensors. This is attributed to the fast, simple, low-cost, and customizable characteristics of the electrospinning process; breathability comes from the porous structure of the electrospun fabrics and wear comfort and conformal attachment brought by the thin and soft characteristics of the electrospun fabrics. Moreover, good attachment of the electrospun fabrics may lead to better signal monitoring quality and device stability, which helps to avoid the interference of the wearing of e-skin to human daily activities and avoid the interference of human daily activities to the performance of e-skin. Apart from the electrospinning e-skin, substrate-free or tattoo-like e-skin has also made great progress because it is easier to achieve breathable and senseless attachment to the skin. However, facing the daily long-term wearing, they must overcome the reduction of durability and stability that comes from the lack of substrate, as well as overcome the challenges of processing difficulty and device reliability.

For the breathability of e-skin, in Sects. 2 and 3, we compared them in Tables 1 and 2, respectively. During comparisons, we found that there are various evaluation methods for the breathability of e-skin, and the standards are not uniform. Although a considerable amount of work has been done to evaluate breathability through water vapor permeability, experimental conditions for measuring the breathability of e-skin by water vapor also varied. For example, some work uses ambient temperature and pressure to test vapor permeability, while others facing wearable applications, use the body temperature to test vapor permeability. Besides, the environment humidity, test equipment, and recording time are different. This prevents the evaluation of breathability and parallel comparison of e-skins with different breathability. Moreover, for parameters used to evaluate the breathability of the e-skin, such as sweat transport rate (STR) and vapor transmission rate (VTR), at present, there are more VTR tests for breathable e-skin than STR tests. This is partly due to the difficulty of carrying out STR tests. However, STR may be a better parameter to reflect the breathability of the e-skin, which is suitable for daily long-term wear application scenarios. Generally speaking, for film breathability tests, ISO 9239–1995 is the widely recognized test standard that has been referred to by many researchers. At present, increasing the porosity of the substrate is a widely used method to improve the breathability of the e-skin. The subsequent research on the layout of components and circuits on the substrate as well as increasing the porosity while ensuring the reliability and stability of the e-skin are the current research focus.

It can be seen from the above sections that novel low-dimensional nanomaterials such as graphene, carbon nanotubes, and MXene play an important role in the study of breathable e-skin due to their excellent performances [5, 13, 31, 36, 37, 49, 55, 67, 68, 74, 75, 78, 89]. Apart from their advantages, the common advantages of the novel low-dimensional nanomaterials in the application of breathable e-skin include: (1) They have many physical and chemical advantages when used as electrode conductive materials, including the large specific surface area and flexibility suitable for the adhesion to a flexible interface like human skin, which helps reduce the electrode–skin contact impedance and ensuring high-quality signal acquisition; (2) easy to realize liquid phase processing, suitable for dip coating, spraying, and other simple processes to fabricate breathable e-skin; (3) good electrical properties, suitable for acting as conductive materials of e-skin electrodes, sensors, and systems; (4) due to the progress of preparation technology, their costs have been reduced compared with traditional precious metal materials, etc., both in terms of materials and fabricating methods; (5) their biocompatibility and stability are consistent with daily long-term skin wear requirements. In conclusion, with the development of related materials, the breathable e-skin based on novel low-dimensional nanomaterials is expected to develop vigorously in the next period. Based on the analysis of various advanced breathable e-skin in recent years, we believe that the future breathable e-skins may have the following three important development directions: (1) The breathable e-skins are becoming more multifunctional to monitor, process, transfer, or even analyze more kinds of signals. (2) The breathable e-skins are becoming more systematic. With a higher integration level, the e-skin can complete a variety of tasks in situ and become an all-in-one system. (3) The breathable e-skins are becoming smarter. Their information processing efficiency will be greatly improved and the function of signal diagnosis can be realized. In the following sections, we are no longer limited to the examples of the breathable e-skin, but take the recently advanced e-skin for physiological signal monitoring as examples to forecast the three development directions and discuss their main challenges, respectively.

### Breathable e-Skins are Becoming Multifunctional

**Fig. 6:**
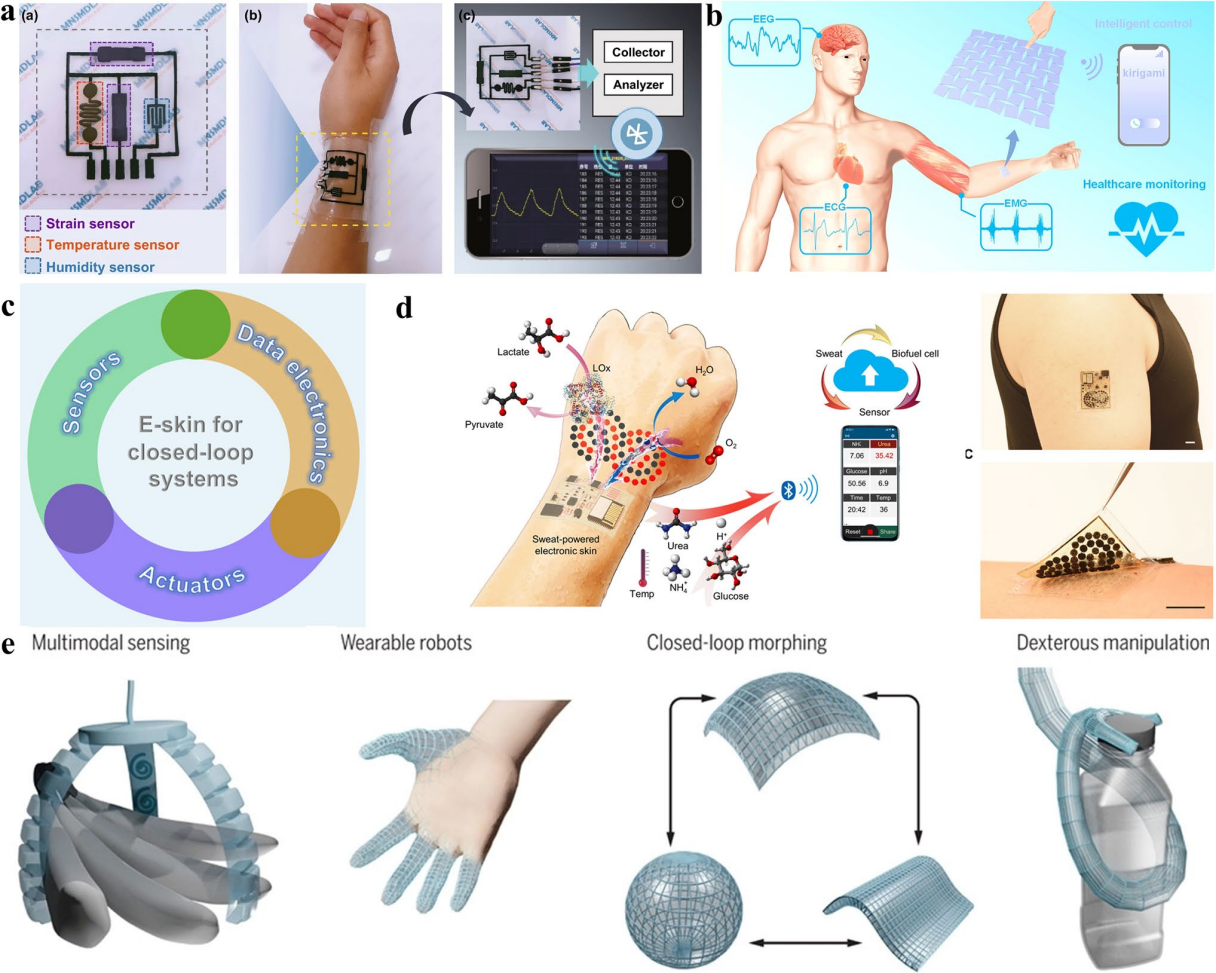
More multifunctional, integrated, and smarter e-skin. **a** Photograph of the e-skin containing strain sensors, temperature sensor, and humidity sensor. Reproduced with permission from Ref. [94]. Copyright 2021 Springer. **b** Self-powered e-skin for intelligent control. Reproduced with permission from Ref. [95]. Copyright 2022 American Chemical Society. **c** Closed-loop e-skin systems. Reproduced with permission from Ref. [96]. Copyright 2019 American Chemical Society. **d** Perspiration-powered e-skin for multiplexed wireless sensing. Reproduced with permission from Ref. [97]. Copyright 2019 AAAS. **e** Potential capabilities and technologies that could be achieved with e-skins. Reproduced with permission from Ref. [8]. Copyright 2020 AAAS

For a variety of physiological signals that require daily monitoring, breathable e-skin can be used as a platform to carry multiple physiological signal sensors and electrodes, which can greatly improve the monitoring efficiency. It can assess the health status of people or give early warning of diseases by analyzing multiple physiological signals comprehensively, which is beneficial to improve the accuracy and reliability of the analysis made by the breathable e-skin. However, as e-skins are becoming multifunctional, some key problems are gradually exposed. Lin et al. proposed that the e-skin with more functions should consider the reasonable layout of modules on the e-skin platform to avoid mutual signal interference [94] (Fig. 6a). Besides, in daily long-term wearing applications, the interference of human daily activities and the external environment to the monitored signals should be fully considered in the design of multifunctional e-skin [95] (Fig. 6b). With the solving of these problems, the multifunctional breathable e-skin will play a promising role in the monitoring of multiple physiological signals, health management, and disease warning in daily life.

### Breathable e-Skins are Becoming Systematic

With the improvement of the function of the breathable e-skin system, its integration degree is also gradually improving. In this process, many advanced systematic modes including “closed-loop system,” “all-in-one system,” and “self-powered system,” etc., have been developed [96] (Fig. 6c). Some of them can achieve complete physiological signal acquisition-transport-processing capability, some can send the signal to the back end for further processing and give feedback to the wearer, and some can acquire the required energy during wearing [97] (Fig. 6d). However, the systematization of the breathable e-skin is still in a relatively early stage. For example, it is still difficult to achieve a small volume e-skin with a complete in-situ signal monitoring-processing-feedback close-loop. Efficient signal processing largely depends on the system with a larger volume and higher computing power at the back end. In addition, power consumption is also an important obstacle to the real-time processing and wireless transmission of the signals monitored by the e-skin. With the gradual solution of these problems, highly integrated and systematic e-skin will be able to realize the daily health management and early warning functions similar to doctors and play an important role in human–machine interaction.

### Breathable e-Skins are Becoming Smarter

The human body produces a large number of physiological signals all the time. Since many health problems and diseases are not necessarily detected by physiological signal monitoring in a specific period, a breathable e-skin suitable for daily long-term physiological signal monitoring has huge advantages over simple hospital checkups. However, in the face of massive physiological signal analysis requirements, the information processing efficiency and power consumption of traditional e-skins are not adequate. To resolve such contradictions, some intelligent algorithms with high information processing efficiency and low power consumption have been developed to give intelligent detection or signal processing capability to wearable e-skins [8, 93] (Fig. 6e). In contrast, the problems that need to be solved during the development of intelligent e-skin systems are emerging. For example, due to the limitation of data volume and power consumption, it is still a challenge to realize real-time signal processing by intelligent algorithms. Whether it is possible or necessary to rapidly process and feedback physiological information at every moment is a problem to be considered. In addition, if in-situ processing or diagnosis of physiological signals is realized by means such as edge computation, whether the obtained conclusions meet the requirements of medical diagnosis remains to be studied. Moreover, to apply intelligent algorithms in wearable e-skins, it is necessary to know how to simultaneously reduce power consumption and improve computing efficiency. With the continuous solution of these problems and the development of the intelligent degree of the breathable e-skin, it may be possible to realize the daily-use breathable e-skin with medical diagnostic ability.

## Conclusion

In this paper, we systematically review the recent development of breathable e-skins for daily physiological monitoring. The typical breathable e-skins are introduced and discussed in detail by dividing them into electrodes, sensors, and systems, and we sort out their design ideas, manufacturing processes, performances, applications, and state their advantages in long-term physiological signal monitoring. Moreover, the new development directions and challenges of the breathable e-skin are discussed and prospected. It is believed that with the continuous development of breathable e-skin, a multifunctional, highly integrated, and intelligent breathable e-skin with the complete and efficient “signal sensing-transmission-diagnosis” capability will be developed and will play a critical role in daily long-term healthcare like a real doctor.
